# The nutritional levels and status of vitamins among children in Henan, China

**DOI:** 10.3389/fnut.2025.1657153

**Published:** 2025-09-26

**Authors:** Xiaojuan Li, Nan Chen, Lei Guo, Yusheng Luan, Zhipeng Jin, Junmei Yang, Tiewei Li

**Affiliations:** Department of Clinical Laboratory, Zhengzhou Key Laboratory of Children’s Infection and Immunity, Children’s Hospital Affiliated to Zhengzhou University, Henan Children’s Hospital, Zhengzhou Children’s Hospital, Zhengzhou, China

**Keywords:** children, vitamin status, age, sex, season

## Abstract

**Background:**

Vitamins are vital for children’s health, and deficiencies can cause disorders, compromising quality of life and survival. This study systematically assesses multivitamin levels, nutritional status, and associated factors in children from Henan, China. It aims to provide robust evidence to improve pediatric nutrition and inform policies for local governments and institutions.

**Methods:**

This retrospective study analyzed data from 1,995 healthy children who underwent routine physical examinations between March 1, 2022, and May 20, 2024. General clinical information and vitamin test results, including vitamins A, D, B1, B2, B3, B5, B7, and C, were retrieved from electronic medical records. Participants were categorized into four age groups: under 3 years, 3–5 years, 6–11 years and 12–18 years. Seasonal classification comprised spring, summer, autumn, and winter.

**Results:**

The cohort consisted of 1,185 males and 810 females. Age distribution included 248 children under 3 years, 743 children aged 3 to 5 years, 883 children aged 6 to 11 years and 121 children aged 12 years or older. Analysis of vitamin insufficiency revealed the highest insufficiency rates for vitamin B7 (58.8%), followed by vitamin D (28.5%), A (28.0%), B1 (11.8%), C (9.1%), E (2.2%), and B3 (0.1%). Notable sex-specific differences were identified in vitamin D, E, B2, B5, and C levels. Age-dependent variations were observed for vitamin A, D, E, B1, B2, B3, B5, B7, and C, while seasonal fluctuations impacted vitamin A, D, E, B1, B3, B5, B7, and C. Sex-based analysis indicated a higher prevalence of vitamin A and C insufficiencies in males and a greater incidence of vitamin D insufficiency in females. Preschool children exhibited the highest vitamin A insufficiency rates, whereas adolescent aged children demonstrated the highest insufficiencies in vitamin D, E, B1, B7, and C. Seasonal analysis revealed increased vitamin A and C insufficiencies during summer, heightened vitamin D and B7 insufficiencies in winter, vitamin E insufficiencies in spring, and vitamin B1 deficiencies in autumn.

**Conclusion:**

A high prevalence of vitamin insufficiencies, particularly in vitamins B7, D, A, and C, was observed among children in Henan, China, with variation rates associated with sex, age, and season.

## Introduction

Vitamins are essential micronutrients indispensable for sustaining human health (1). They are classified into water-soluble vitamins (including vitamin C and B-complex vitamins) and fat-soluble vitamins (such as vitamins A, D, E, and K). These nutrients are integral to numerous physiological processes, including energy metabolism, antioxidant defense, immune modulation, and blood coagulation. Vitamin deficiencies can precipitate a range of adverse health outcomes, including compromised immune function, skeletal abnormalities, and cognitive impairments (2–6).

Childhood vitamin insufficiencies/deficiencies represent a significant global public health concern. Current evidence indicates that approximately 25% of children worldwide experience subclinical vitamin A (VA) deficiency, with nearly 250 million children annually at risk of blindness due to inadequate VA intake (7). In China, the prevalence of pediatric vitamin deficiencies, particularly VA and vitamin D (VD), has shown a rising trend. A cross-sectional survey conducted in Jiangsu Province between 2016 and 2017 reported a 0.8% prevalence of VA deficiency among children, with an additional 15.8% classified as marginally deficient (8). Notably, geographic disparities persist, with rural children displaying a significantly elevated risk of VA deficiency compared to their urban counterparts (9). VD deficiency is similarly concerning, affecting 23.2% of children with deficient levels and 54.2% with insufficient levels. The pediatric VD status is influenced by a range of factors, including sex, age, geographical location, duration of sunlight exposure, and maternal education (10). In northern China, children exhibit increased rates of VD deficiency during winter due to reduced ultraviolet radiation exposure (9). Moreover, deficiencies in B vitamins among children constitute a substantial global public health challenge (11). As children age, their physiological demand for B vitamins increases; however, dietary practices in many households fail to meet these requirements, resulting in significant nutritional gaps (12).

Currently, evidence suggests an age-dependent increase in the prevalence of vitamin deficiencies among children (13). Children’s nutritional status directly impacts their future health and developmental potential. However, comprehensive investigations into the vitamin nutritional levels and status of children in Henan, China are still lacking. This study aims to systematically assess the nutritional status of various vitamins among children in Henan, China and identify key influencing factors through epidemiological surveys. The outcomes are intended to inform public health policy, support clinicians in identifying high-risk groups, and guide the development of targeted intervention strategies. Additionally, the findings will provide practical insights into pediatric healthcare and nutrition, promoting a more evidence-based and standardized approach to managing child health.

## Materials and methods

### Study design and population

A retrospective study was conducted at Henan Provincial Children’s Hospital (Children’s Hospital Affiliated to Zhengzhou University). Henan Provincial Children’s Hospital is a tertiary hospital located in a major city in Henan, China. It is designated as a National Regional Medical Center for Children and is recognized as the Henan Provincial Children’s Medical Center. Thanks to the country’s comprehensive child health insurance system, children from diverse socioeconomic backgrounds can receive treatment at this center. As one of the largest children’s hospitals in Henan, China, it serves patients from Henan Province, making its patient population a representative sample of the pediatric population in Henan, China. From March 1, 2022, to May 20, 2024, 1,995 healthy children who underwent routine physical examinations at the Department of Child Health Care and Health Management Center were enrolled in this study. The inclusion criteria encompassed: (1) age below 18 years; (2) availability of complete electronic medical records containing age, sex, and vitamin assay results. Exclusion criteria comprised: (1) diagnosis of infectious diseases; (2) history of congenital anomalies; (3) presence of growth and developmental disorders; (4) hematological diseases or dysfunctions of the liver, kidneys, or heart.

The selection of vitamins was based on their established roles in child development and emerging regional concerns regarding suboptimal micronutrient status, particularly in areas undergoing dietary transition. While vitamins such as A, D, and C are widely recognized for their clinical significance, other B-vitamins (e.g., B1, B2, B3, B5, B7) were included to provide a comprehensive nutritional overview and support public health surveillance, given limited contemporary data in this population. It should be noted that biochemical deficiency does not invariably correspond to overt clinical disease; rather, it identifies populations at potential risk or with suboptimal status.

The study protocol adhered to the principles of the Declaration of Helsinki and was approved by the Ethics Review Committee of Henan Children’s Hospital (Approval No. 2022-K-L045). As the study employed anonymized retrospective data obtained during standard clinical practice, the requirement for informed consent was waived, as verified by the Ethics Review Board of Henan Children’s Hospital (Approval No. 2022-K-L045).

### Data collection

Demographic data and laboratory parameters were systematically extracted from the electronic medical record system for children undergoing routine health examinations. Demographic variables included age, sex, examination date, and clinical diagnoses. The primary laboratory indicators encompassed vitamin assay results, specifically targeting retinol (VA), 25-(OH)-vitamin D2, 25-(OH)-vitamin D3, vitamin E (VE), vitamin B1 (VB1), vitamin B2 (VB2), vitamin B3 (VB3), vitamin B5 (VB5), vitamin B7 (VB7), and vitamin C (VC). Total VD level was calculated by 25-(OH)-vitamin D2 plus 25-(OH)-vitamin D3. Serum vitamin levels were determined using the ultra-performance liquid chromatography–tandem mass spectrometry (UPLC-MS/MS) system (Waters Corp, Milford, MA). Sample preparation was performed following the manufacturer’s protocol outlined in the commercial reagent kit (Shanghai Kehua Biological Technology Co., Ltd.).

### Operational definitions

1) Age Stratification

Children under 18 years were stratified into three developmental stages:

(a) Infancy/Toddler Period: < 3 years.
(b) Preschool Age: 3–5 years.
(e) School Age: 6 to 11 years.
(f) Adolescent age: ≥ 12 years

2) Seasonal Classification

The calendar year was segmented into four meteorological seasons:

(a) Spring: March–May.
(b) Summer: June–August.
(c) Autumn: September–November.
(d) Winter: December–February of the following year.

3) Vitamin insufficiency criteria

According to the Chinese Expert Consensus on Clinical Applications of VA and VD in Children (2024) (14):

(i) VA (retinol) insufficiency: < 300 ng/mL.
(ii) VD insufficiency: < 20 ng/mL.

For vitamins without established guidelines, insufficiency thresholds were determined based on reference ranges from Mayo Clinic, Labcorp, and Quest Diagnostics:

(a) VE insufficiency: < 3.8 μg/mL (Mayo Clinic).
(b) VB1 insufficiency: < 2.12 ng/mL (Quest Diagnostics).
(c) VB2 insufficiency: < 1 ng/mL (Mayo Clinic).
(d) VB3 insufficiency: < 5.2 ng/mL (Labcorp).
(e) VB5 insufficiency (Mayo Clinic):

(i) Ages 0–10 years: < 3.45 ng/mL.
(ii) Ages > 10 years: < 37 ng/mL.

(f) VB7 insufficiency (Mayo Clinic):

(i) iAges < 12 years: < 0.1 ng/mL.
(ii) Ages ≥ 12 years: < 0.22 ng/mL

(g) VC insufficiency: < 4 μg/mL (Mayo Clinic).

### Statistical analysis

All statistical analyses were performed using SPSS version 24.0. Continuous variables with non-normal distribution were expressed as median values along with interquartile ranges (25th–75th percentiles) and compared using the Mann–Whitney U test. Categorical variables were presented as numerical counts (percentages) and analyzed using the chi-square test. A two-tailed *p*-value < 0.05 was considered statistically significant.

## Results

### Study population characteristics

Between March 1, 2022, and May 20, 2024, a total of 1,995 pediatric patients who underwent routine health examinations were included in this study. As outlined in Table 1, the cohort comprised 1,185 males (59.4%) and 810 females (40.6%). The age distribution included 248 infants/toddlers (aged < 3 years, 12.4%), 743 preschool-aged children (aged 3 to 5 years, 37.2%), 883 school-aged children (aged 6 to 11 years, 44.3%) and 121 adolescents (aged ≥ 12 years, 6.1%). The seasonal distribution of health examinations was as follows: 500 examinations conducted in spring (25.1%), 775 in summer (38.8%), 230 in autumn (11.5%), and 490 in winter (24.6%).

**Table 1:** Basic characteristics of study subjects.

Variables	Total number (*n* = 1,995)
Sex	
Male, *n* (%)	1,185 (59.4%)
Female, *n* (%)	810 (41.0%)
Age	
< 3 years, *n* (%)	248 (12.4%)
3–5 years, *n* (%)	743 (37.2%)
6–11 years, *n* (%)	883 (44.3%)
≥ 6 years *n* (%)	121 (6.1%)
Season	
Spring, *n* (%)	500 (25.1%)
Summer, *n* (%)	775 (38.8%)
Autumn, *n* (%)	230 (11.5%)
Winter, *n* (%)	490 (24.6%)

### Vitamin levels and nutritional status of healthy examined children

As presented in Table 2, the median concentrations (25th percentile, 75th percentile) for VA, VD, VE, VB1, VB2, VB3, VB5, VB7, and VC were as follows: VA: 349.8 (293.7, 414.2) ng/mL; VD: 25.2 (19.0, 32.0) ng/mL; VE: 7.0 (5.7, 8.4) μg/mL; VB1: 3.4 (2.6, 4.7) ng/mL; VB2: 9.2 (6.4, 13.0) ng/mL; VB3: 32.8 (21.9, 50.9) ng/mL; VB5: 52.6 (42.2, 72.0) ng/mL; VB7: 0.085 (0.043, 0.137) ng/mL; and VC: 11.3 (7.5, 15.4) μg/mL. Analysis of vitamin nutritional status revealed that VB7 exhibited the highest insufficiency rate at 58.8%, followed by VD (28.6%), VA (28.0%), VB1 (11.8%), VC (9.1%), VE (2.2%), and VB3 (0.1%). Notably, the insufficiency rates for VB2 and VB5 among the healthy pediatric population were both 0% (Supplementary Table 1).

**Table 2:** Serum vitamins levels of healthy examined children.

Variables	Concentration
VA (ng/mL)	349.8 (293.7, 414.2)
VD (ng/mL)	25.2 (19.0, 32.0)
VE (μg/mL)	7.0 (5.7, 8.4)
VB1 (ng/mL)	3.4 (2.6, 4.7)
VB2 (ng/mL)	9.2 (6.4, 13.0)
VB3 (ng/mL)	32.8 (21.9, 50.9)
VB5 (ng/mL)	52.6 (42.2, 72.0)
VB7 (ng/mL)	0.085 (0.043, 0.137)
VC (μg/mL)	11.3 (7.5, 15.4)

VA, vitamin A, VD, vitamin D, VE, vitamin E, VB1, vitamin B1, VB2, vitamin B2, VB3, vitamin B3, VB5, vitamin B5, VB7, vitamin B7, VC, vitamin C.

### Sex-specific variations in pediatric vitamins levels and nutritional status

Male participants demonstrated significantly higher peripheral blood levels of VD and VB5 compared to their female counterparts. In contrast, female participants exhibited markedly elevated levels of VE, VB2, and VC. No statistically significant differences between sex were observed for VA, VB1, VB3, and VB7 (Table 3). Analysis of vitamin nutritional status indicated a significantly greater prevalence of VC insufficiency among male children, whereas VD insufficiency was significantly more prevalent among female children (Supplementary Table 2).

**Table 3:** Sex-specific vitamins levels.

Variables	Male (*n* = 1,185)	Female (*n* = 810)	*p*
VA (ng/mL)	346.9 (290.4, 413.9)	354.9 (297.4, 417.5)	0.120
VD (ng/mL)	25.6 (19.7, 32.1)	24.6(17.7, 31.8)	0.035
VE (μg/mL)	6.8 (5.6, 8.3)	7.1 (5.9, 8.7)	< 0.001
VB1 (ng/mL)	3.4 (2.6, 4.6)	3.4 (2.6, 4.8)	0.562
VB2 (ng/mL)	8.9 (6.3, 12.4)	9.6 (6.7, 13.9)	< 0.001
VB3 (ng/mL)	33.3 (22.1, 51.4)	32.3 (21.8, 50.2)	0.384
VB5 (ng/mL)	53.5 (42.9, 73.2)	51.5 (40.8, 70.8)	0.015
VB7 (ng/mL)	0.083 (0.040, 0.136)	0.087 (0.046, 0.139)	0.139
VC (μg/mL)	10.9 (7.0, 15.2)	11.6 (7.8, 15.6)	0.014

VA, vitamin A, VD, vitamin D, VE, vitamin E, VB1, vitamin B1, VB2, vitamin B2, VB3, vitamin B3, VB5, vitamin B5, VB7, vitamin B7, VC, vitamin C.

### Age-specific variations in pediatric vitamin levels and nutritional status

Serum concentrations of VA, VD, VE, and B-complex vitamins (B1, B2, B3, B5, B7), as well as VC, exhibited significant age-dependent variations among children. VA levels were notably lowest within the 3–5-year age group, while VD, VE, and all analyzed B vitamins (B1, B2, B3, B5, B7), as well as VC, demonstrated progressive declines with increasing age (Table 4). Vitamin nutritional status analysis revealed distinct age-related insufficiency patterns. The highest prevalence of VA insufficiency (31.1%) occurred in the 3–5-year group. In contrast, the insufficiency rates of VD, VE, VB1, VB7, and VC displayed significant positive correlations with age, peaking at 60.3, 5.0, 33.9, 100.0, and 24.8%, respectively, among children aged ≥ 12 years (Supplementary Table 3).

**Table 4:** Age-specific vitamins levels.

Variables	< 3 year (*n* = 248)	3–5 year (*n* = 743)	6–11 year (*n* = 883)	≥ 12 year (*n* = 121)	*p*
VA (ng/mL)	353.3 (290.4, 411.4)	340.0 (284.5, 396.5)	350.9 (293.4, 422.2)	397.0 (346.5, 470.3)^cef^	< 0.001
VD (ng/mL)	38.0 (32.6, 46.2)	28.3 (22.7, 33.0)^a^	21.4 (16.5, 26.5)^bd^	17.1 (12.3, 22.5)^cef^	< 0.001
VE (μg/mL)	8.2 (6.8, 10.1)	7.2 (6.0, 8.6)^a^	6.6 (5.45, 8.0)^bd^	6.0 (5.1, 7.4)^cef^	< 0.001
VB1 (ng/mL)	5.8 (3.9, 8.6)	3.6 (2.7, 4.7)^a^	3.2 (2.5, 4.1)^bd^	2.5 (1.9, 3.3)^cef^	< 0.001
VB2 (ng/mL)	13.9 (9.5, 19.4)	9.5 (6.9, 13.1)^a^	8.3 (5.9, 11.7)^bd^	6.9 (5.5, 9.2)^cef^	< 0.001
VB3 (ng/mL)	45.8 (26.6, 74.6)	34.0 (22.7, 50.9)^a^	30.4 (20.8, 45.8)^bd^	31.0 (17.8, 49.1)^cef^	< 0.001
VB5 (ng/mL)	89.3 (52.3, 115.6)	56.9 (46.3, 72.8)^a^	46.6 (38.1, 58.8)^bd^	41.9 (34.6, 52.4)^cef^	< 0.001
VB7 (ng/mL)	0.136 (0.066, 0.212)	0.091 (0.045, 0.141)^a^	0.077 (0.039, 0.122)^bd^	0.072 (0.041, 0.112)^cef^	< 0.001
VC (μg/mL)	14.0 (10.3, 17.8)	12.0 (8.1, 15.9)^a^	10.3 (6.8, 14.4)^bd^	8.1 (4.0, 11.7)^cef^	< 0.001

VA, vitamin A, VD, vitamin D, VE, vitamin E, VB1, vitamin B1, VB2, vitamin B2, VB3, vitamin B3, VB5, vitamin B5, VB7, vitamin B7, VC, vitamin C.

^a^*P* < 0:05 for 3–5 year vs. < 3 year. ^b^*P* < 0:05 for 6–11 year vs. < 3 year. ^c^*P* < 0:05 for ≥ 12 years vs. < 3 year. ^d^*P* < 0:05 for 6–11 year vs. 3–5 year. ^e^*P* < 0:05 for ≥ 12 years vs. 3–5 year. ^f^*P* < 0:05 for ≥ 12 years vs. 6–11 year.

### Season-specific variations in pediatric vitamins levels and nutritional status

Analysis of vitamin levels in pediatric subjects undergoing health examinations identified significant seasonal variations in VA, VD, VE, VB1, VB3, VB5, VB7, and VC. VA, VB1, VB5, and VC levels reached their lowest values during summer, while VD and VB7 levels were minimized in winter. VE and VB3 levels were lowest in spring (Table 5). Evaluation of vitamin nutritional status revealed pronounced seasonal disparities in insufficiency rates for VA, VD, VE, and VC. The highest insufficiency rates for VA (32.9%) and VC (13.7%) occurred in summer, whereas VD (42.2%) and VB7 (61.6%) insufficiency rates were most prevalent in winter. Additionally, VE insufficiency reached its peak in spring (4.4%), and VB1 demonstrated the highest insufficiency rate in autumn (16.5%) (Supplementary Table 4).

**Table 5:** Season-specific vitamins levels.

Variables	Spring (*n* = 500)	Summer (*n* = 775)	Autumn (*n* = 230)	Winter (*n* = 490)	*p*
VA (ng/mL)	344.6 (294.7, 402.9)	341.6 (282.8, 422.6)	348.7 (299.3, 400.1)	370.0 (308.6, 432.3)^cef^	< 0.001
VD (ng/mL)	24.4 (17.9, 31.8)	27.1 (21.5, 32.8)^a^	25.4 (20.5, 32.7)^b^	22.0 (15.2, 29.7)^cef^	< 0.001
VE (μg/mL)	6.6 (5.4, 8.0)	7.5 (6.3, 8.9)^a^	6.7 (5.4, 8.3)^d^	6.7 (5.6, 8.1)^e^	< 0.001
VB1 (ng/mL)	3.7 (2.7, 5.0)	3.3 (2.6, 4.4)^a^	3.3 (2.5, 4.6)^b^	3.5 (2.5, 5.0)^e^	0.004
VB2 (ng/mL)	9.2 (6.3, 12.6)	9.3 (6.5, 13.1)	9.6 (6.4, 13.8)	8.9 (6.4, 13.2)	0.559
VB3 (ng/mL)	29.6 (20.0, 44.6)	34.6 (23.4, 51.8)^a^	34.3 (19.3, 55.2)	33.1 (22.8, 52.0)^c^	< 0.001
VB5 (ng/mL)	53.1 (42.4, 73.7)	50.5 (40.7, 70.3)^a^	57.3 (44.2, 76.4)^d^	53.6 (42.4, 71.9)^e^	0.008
VB7 (ng/mL)	0.094 (0.052, 0.145)	0.084 (0.045, 0.132)	0.081 (0.042, 0.144)	0.079 (0.030, 0.135)^c^	0.025
VC (μg/mL)	12.1 (7.6, 16.9)	9.8 (6.2, 13.4)^a^	10.5 (7.3, 14.1)^b^	13.3 (9.7, 16.8)^cef^	< 0.001

VA, vitamin A, VD, vitamin D, VE, vitamin E, VB1, vitamin B1, VB2, vitamin B2, VB3, vitamin B3, VB5, vitamin B5, VB7, vitamin B7, VC, vitamin C.

^a^*P* < 0:05 for 3–5 year vs. < 3 year; ^b^*P* < 0:05 for 6–11 year vs. < 3 year; ^c^*P* < 0:05 for ≥ 12 years vs. < 3 year; ^d^*P* < 0:05 for 6–11 year vs. 3–5 year; ^e^*P* < 0:05 for ≥ 12 years vs. 3–5 year; ^f^*P* < 0:05 for ≥ 12 years vs. 6–11 year.

## Discussion

Vitamins are fundamental nutrients essential for maintaining normal physiological functions, particularly during key stages of growth and development in childhood. Adequate vitamin intake is crucial for both physical and cognitive maturation. Vitamin deficiencies in children constitute a major global public health issue, exacerbated by the rapid progression of globalization and urbanization, particularly in low- and middle-income countries. Insufficient vitamin intake during childhood is associated with stunted growth, compromised immunity, and impaired cognitive abilities. Deficiencies in VA are linked to a range of visual impairments, including night blindness, xerophthalmia, and severe complications such as corneal ulceration and potential blindness (15). According to the World Health Organization, approximately 200 million children worldwide experience VA deficiency, directly affecting growth and immune function (16). VD deficiency is associated with skeletal abnormalities, including rickets (17), and recent studies have identified correlations between VD deficiency and increased risks of infectious diseases, cardiovascular conditions, and neurological disorders (5, 18, 19). Additionally, VE deficiency is linked to neurological impairments and immune dysfunction (20), while B vitamin deficiencies are implicated in various cardiovascular, neurological, and dermatological conditions (21–24).

Henan Province, situated in Henan, China, is the cradle of Huaxia civilization with over 3,200 years of recorded history. It served as the cultural, economic, and political center of China until approximately 1,000 years ago. Henan has a substantial child population; according to data from the Henan Provincial Government at the end of 2024, children under 15 years old numbered 20.71 million, constituting 21.2% of the total population. Furthermore, Henan Provincial Children’s Hospital, as the sole National Regional Medical Center for Children in Henan, China, provides services to children from Henan and neighboring provinces. However, the prevalence of vitamin deficiency disorders among children is increasing, and children’s nutritional status directly impacts their future health and developmental potential. Therefore, it is imperative to investigate the vitamin nutritional levels and status of children in Henan, China.

This study provides the first comprehensive assessment of multiple vitamin levels and nutritional status among children in Henan, China. It found that female children exhibited a higher prevalence of VD insufficiency. This disparity may be attributed to reduced sunlight exposure, thereby limiting endogenous VD synthesis. In contrast, female children typically present with higher levels of VE, VB2, VB5, and VC, accompanied by lower insufficiency rates compared to males. Sex-specific variations in vitamin requirements are evident; adolescent males generally exhibit higher energy and vitamin demands, which are attributed to accelerated growth rates, increased weight gain, and higher physical activity levels. Moreover, dietary patterns differ between sex, with females tending to adopt more balanced nutritional practices. In contrast, males are more likely to engage in selective eating, increasing the risk of micronutrient deficiencies.

Vitamin requirements also vary significantly across developmental stages. Infants necessitate increased VD intake to support proper skeletal growth, while adolescents require higher levels of VA and VC to facilitate immune system maturation (25). Changes in dietary habits, nutrient consumption, and physiological demands with age can result in vitamin deficiencies, adversely affecting health outcomes (26). The analysis identified distinct age-related trends, with levels of VD, VE, VB1, VB2, VB3, VB5, VB7, and VC progressively declining as age increases. Correspondingly, insufficiency rates for VD, VE, VB1, VB7, and VC rise with age, reaching their lowest levels in infants under 2 years and peaking in adolescent age over 12 years. This pattern likely reflects optimal nutrition during infancy, characterized by breastfeeding, fortified formulas, and routine VD supplementation to prevent rickets, contrasted with decreased dietary regulation and reduced outdoor activities as children grow older.

Seasonal fluctuations in vitamin levels arise from a complex interplay of factors, including sunlight exposure, dietary patterns, and physiological adaptations. VD levels exhibit pronounced seasonal variability, with deficiency risk significantly increasing during winter due to reduced daylight and lower UVB radiation (27). This study corroborates these findings, identifying the lowest VD levels and the highest insufficiency rates during winter. Seasonal variations also significantly affect VA, VE, VB1, VB3, VB5, VB7, and VC levels. Data analysis reveals that insufficiency rates for VA and VC peak during summer, while VE insufficiency is most prevalent in spring, and VC insufficiency rates increase in summer. These seasonal differences are likely influenced by the variable availability of fresh fruits and vegetables throughout the year (28).

This comprehensive analysis identifies significant associations between vitamin insufficiency rates and factors such as sex, age, and seasonal variations. However, several limitations should be considered. First, the single-center design may restrict the generalizability of our findings, and future multicenter studies are warranted for validation. Second, the lack of data on dietary habits, lifestyle factors, and socioeconomic status may introduce residual confounding. Furthermore, the limited sample size in certain subgroups—particularly within the broadly defined 12-18-year age group encompassing diverse pubertal stages—restricted finer age stratification and may have obscured more nuanced developmental trends. Additionally, the assessment of thiamine status relied solely on total blood thiamine levels rather than more sensitive functional assays, which may reduce the sensitivity to detect functional thiamine insufficiency.

## Conclusion

In conclusion, this study reveals a high prevalence of vitamin insufficiencies, particularly vitamins B7, D, A, and C among children in Henan, China, with variations significantly associated with sex, age, and season. These findings provide critical evidence to guide public health policy formulation and support the implementation of targeted nutritional interventions, such as age-specific supplementation and seasonal dietary recommendations, to improve pediatric health outcomes in this population.

## Data Availability

The original contributions presented in the study are included in the article/Supplementary material, further inquiries can be directed to the corresponding authors.
