# Supplementary material for: The nutritional levels and status of vitamins among children in Henan, China
Source: Front Nutr. 2025 Sep 26;12:1657153. doi: 10.3389/fnut.2025.1657153 (PMC12510813; doi:10.3389/fnut.2025.1657153)
Supplement: Supplementary file 1 [file Table_1.DOCX]

Supplemental Table 1. Vitamins nutritional status of healthy examined children.

| Variables | Total number (n = 1,995) |
| --- | --- |
| VA status |  |
| VA sufficiency, n (%) | 1437 (72.0%) |
| VA insufficiency, n (%) | 558 (28.0%) |
| VD status |  |
| VD sufficiency, n (%) | 1,425 (71.4%) |
| VD insufficiency, n (%) | 570 (28.6%) |
| VE status |  |
| VE sufficiency, n (%) | 1,952 (97.8%) |
| VE insufficiency, n (%) | 43 (2.2%) |
| VB1 status |  |
| VB1 sufficiency, n (%) | 1,759 (88.2%) |
| VB1 insufficiency, n (%) | 236 (11.8%) |
| VB2 status |  |
| VB2 sufficiency, n (%) | 1,995 (100.0%) |
| VB2 insufficiency, n (%) | 0 (0) |
| VB3 status |  |
| VB3 sufficiency, n (%) | 1,993 (99.9%) |
| VB3 insufficiency, n (%) | 2 (0.1%) |
| VB5 status |  |
| VB5 sufficiency, n (%) | 1,995 (100.0%) |
| VB5 insufficiency, n (%) | 0 (0) |
| VB7 status |  |
| VB7 sufficiency, n (%) | 822 (41.2%) |
| VB7 insufficiency, n (%) | 1,173 (58.8%) |
| VC status |  |
| VC sufficiency, n (%) | 1,813 (90.9%) |
| VC insufficiency, n (%) | 182 (9.1%) |

Abbreviations: VA, vitamin A, VD, vitamin D, VE, vitamin E, VB1, vitamin B1, VB2, vitamin B2, VB3, vitamin B3, VB5, vitamin B5, VB7, vitamin B7, VC, vitamin C.

Supplemental Table 2. Sex-specific vitamins nutritional status

| Variables | Male (n = 1,185) | Female (n = 810) | p |
| --- | --- | --- | --- |
| VA status |  |  | 0.075 |
| VA sufficiency, n (%) | 836 (70.5%) | 601 (74.2%) |  |
| VA insufficiency, n (%) | 349 (29.5%) | 209 (25.8%) |  |
| VD status |  |  | 0.020 |
| VD sufficiency, n (%) | 874 (73.8%) | 551 (68.0%) |  |
| VD insufficiency, n (%) | 311 (26.2%) | 259 (32.0%) |  |
| VE status |  |  |  |
| VE sufficiency, n (%) | 1,157 (97.6%) | 795 (98.1%) | 0.526 |
| VE insufficiency, n (%) | 28 (2.4%) | 15 (1.9%) |  |
| VB1 status |  |  | 0.980 |
| VB1 sufficiency, n (%) | 1,045 (88.2%) | 714 (88.1%) |  |
| VB1 insufficiency, n (%) | 140 (11.8%) | 96 (11.9%) |  |
| VB2 status |  |  | 1.000 |
| VB2 sufficiency, n (%) | 1,185 (100.0%) | 810 (100.0%) |  |
| VB2 insufficiency, n (%) | 0 (0) | 0 (0) |  |
| VB3 status |  |  | 0.242 |
| VB3 sufficiency, n (%) | 1,183 (99.8%) | 810 (100.0%) |  |
| VB3 insufficiency, n (%) | 2 (0.2%) | 0 (0) |  |
| VB5 status |  |  | 1.000 |
| VB5 sufficiency, n (%) | 1,185 (100.0%) | 810 (100.0%) |  |
| VB5 insufficiency, n (%) | 0 (0) | 0 (0) |  |
| VB7 status |  |  | 0.297 |
| VB7 sufficiency, n (%) | 477 (40.3%) | 345 (42.6%) |  |
| VB7 insufficiency, n (%) | 708 (59.7%) | 465 (57.4%) |  |
| VC status |  |  | 0.003 |
| VC sufficiency, n (%) | 1,058 (89.3%) | 755 (93.2%) |  |
| VC insufficiency, n (%) | 127 (10.7%) | 55 (6.8%) |  |

Abbreviations: VA, vitamin A, VD, vitamin D, VE, vitamin E, VB1, vitamin B1, VB2, vitamin B2, VB3, vitamin B3, VB5, vitamin B5, VB7, vitamin B7, VC, vitamin C.

Supplemental Table 3. Age-specific vitamins nutritional status

| Variables | < 3 year  (n = 248) | 3-5 year  (n = 743) | 6-11 year  (n = 883) | ≥ 12 year  (n = 121) | p |
| --- | --- | --- | --- | --- | --- |
| VA status |  |  |  |  | < 0.001 |
| VA sufficiency, n (%) | 180 (72.6%) | 512 (68.9%) | 636 (72.0%) | 109 (90.1%)^cef^ |  |
| VA insufficiency, n (%) | 68 (27.4%) | 231 (31.1%) | 259 (28.0%) | 12 (9.9%)^cef^ |  |
| VD status |  |  |  |  | < 0.001 |
| VD sufficiency, n (%) | 244 (98.4%) | 626 (84.3%)^a^ | 507 (57.4%)^bd^ | 48 (39.7%)^cef^ |  |
| VD insufficiency, n (%) | 4 (1.6%) | 117 (15.7%)^a^ | 376 (42.6%)^bd^ | 73 (60.3%)^cef^ |  |
| VE status |  |  |  |  |  |
| VE sufficiency, n (%) | 245 (98.8%) | 734 (98.8%) | 858 (97.2%) | 115 (95.0%)^e^ | 0.014 |
| VE insufficiency, n (%) | 3 (1.2%) | 9 (1.2%) | 25 (2.8%) | 6 (5.0%)^e^ |  |
| VB1 status |  |  |  |  | < 0.001 |
| VB1 sufficiency, n (%) | 240 (96.8%) | 673 (90.6%)^a^ | 766 (86.7%)^b^ | 80 (66.1%)^cef^ |  |
| VB1 insufficiency, n (%) | 8 (3.2%) | 70 (9.4%)^a^ | 117 (13.3%)^b^ | 41 (33.9%)^cef^ |  |
| VB2 status |  |  |  |  | 1.000 |
| VB2 sufficiency, n (%) | 248 (100.0%) | 743 (100.0%) | 883 (100.0%) | 121(100.0%) |  |
| VB2 insufficiency, n (%) | 0 (0) | 0 (0) | 0 (0) | 0 (0) |  |
| VB3 status |  |  |  |  | 0.471 |
| VB3 sufficiency, n (%) | 248 (100.0%) | 743 (100.0%) | 881 (99.8%) | 121 (100%) |  |
| VB3 insufficiency, n (%) | 0 (0) | 0 (0) | 2 (0.2%) | 0 (0) |  |
| VB5 status |  |  |  |  | 1.000 |
| VB5 sufficiency, n (%) | 248 (100.0%) | 743 (100.0%) | 883 (100%) | 121 (100.0%) |  |
| VB5 insufficiency, n (%) | 0 (0) | 0 (0) | 0 (0%) | 0 (0) |  |
| VB7 status |  |  |  |  | < 0.001 |
| VB7 sufficiency, n (%) | 155 (62.5%) | 337 (45.4%)^a^ | 330 (37.4%)^bd^ | 0 (0)^cef^ |  |
| VB7 insufficiency, n (%) | 93 (37.5%) | 406 (54.6%)^a^ | 553 (62.6%)^bd^ | 121 (100.0%)^cef^ |  |
| VC status |  |  |  |  | < 0.001 |
| VC sufficiency, n (%) | 243 (98.0%) | 692 (93.1%)^a^ | 787 (89.1%)^bd^ | 91 (75.2%)^cef^ |  |
| VC insufficiency, n (%) | 5 (2.0%) | 51 (6.9%)^a^ | 96 (10.9%)^bd^ | 30 (24.8%)^cef^ |  |

Abbreviations: VA, vitamin A, VD, vitamin D, VE, vitamin E, VB1, vitamin B1, VB2, vitamin B2, VB3, vitamin B3, VB5, vitamin B5, VB7, vitamin B7, VC, vitamin C.

^a^P < 0:05 for 3-5 year vs. < 3 year; ^b^P < 0:05 for 6-11 year vs. < 3 year; ^c^P < 0:05 for ≥ 12 years vs. < 3 year; ^d^P < 0:05 for 6-11 year vs. 3-5 year; ^e^P < 0:05 for ≥ 12 years vs. 3-5 year; ^f^P < 0:05 for ≥ 12 years vs. 6-11 year.

Supplemental Table 4. Season-specific vitamins nutritional status

| Variables | Spring  (n = 500) | Summer  (n = 775) | Autumn  (n = 230) | Winter  (n = 490) | p |
| --- | --- | --- | --- | --- | --- |
| VA status |  |  |  |  | < 0.001 |
| VA sufficiency, n (%) | 362 (72.4%) | 520 (67.1%) | 171 (74.3%) | 384 (78.4%)^e^ |  |
| VA insufficiency, n (%) | 138 (27.6%) | 255 (32.9%) | 59 (25.7%) | 106 (21.6%)^e^ |  |
| VD status |  |  |  |  | < 0.001 |
| VD sufficiency, n (%) | 335 (67.0%) | 628 (81.0%)^a^ | 178 (77.4%)^b^ | 283 (57.8%)^cef^ |  |
| VD insufficiency, n (%) | 165 (33.0%) | 147 (19.0%)^a^ | 51 (22.2%)^b^ | 207 (42.2%)^cef^ |  |
| VE status |  |  |  |  | < 0.001 |
| VE sufficiency, n (%) | 478 (95.6%) | 768 (99.1%)^a^ | 225 (97.8%) | 481 (98.2%) |  |
| VE insufficiency, n (%) | 22 (4.4%) | 7 (0.9%)^a^ | 5 (2.2%) | 9 (1.8%) |  |
| VB1 status |  |  |  |  | 0.077 |
| VB1 sufficiency, n (%) | 451 (90.2%) | 684 (88.3%) | 192 (83.5%) | 432 (88.2%) |  |
| VB1 insufficiency, n (%) | 49 (9.8%) | 91 (11.7%) | 38 (16.5%) | 58 (11.8%) |  |
| VB2 status |  |  |  |  | 1.000 |
| VB2 sufficiency, n (%) | 500 (100.0%) | 775 (100.0%) | 230 (100.0%) | 490 (100.0%) |  |
| VB2 insufficiency, n (%) | 0 (0) | 0 (0) | 0 (0) | 0 (0) |  |
| VB3 status |  |  |  |  | 0.369 |
| VB3 sufficiency, n (%) | 500 (100.0%) | 773 (99.7%) | 230 (100.0%) | 490 (100.0%) |  |
| VB3 insufficiency, n (%) | 0 (0) | 2 (0.3%) | 0 (0) | 0 (0) |  |
| VB5 status |  |  |  |  | 1.000 |
| VB5 sufficiency, n (%) | 500 (100.0%) | 775 (100.0%) | 230 (100.0%) | 490 (100.0%) |  |
| VB5 insufficiency, n (%) | 0 (0) | 0 (0) | 0 (0) | 0 (0) |  |
| VB7 status |  |  |  |  | 0.113 |
| VB7 sufficiency, n (%) | 228 (45.6%) | 315 (40.6%) | 91 (39.6%) | 188 (38.4%) |  |
| VB7 insufficiency, n (%) | 272 (54.4%) | 460 (59.4%) | 139 (60.4%) | 302 (61.6%) |  |
| VC status |  |  |  |  | < 0.001 |
| VC sufficiency, n (%) | 465 (93.0%) | 669 (86.3%)^a^ | 210 (91.3%) | 469 (87.6%)^e^ |  |
| VC insufficiency, n (%) | 35 (7.0%) | 106 (13.7%)^a^ | 20 (8.7%) | 21 (4.3%)^e^ |  |

Abbreviations: VA, vitamin A, VD, vitamin D, VE, vitamin E, VB1, vitamin B1, VB2, vitamin B2, VB3, vitamin B3, VB5, vitamin B5, VB7, vitamin B7, VC, vitamin C.

^a^P < 0:05 for 3-5 year vs. < 3 year; ^b^P < 0:05 for 6-11 year vs. < 3 year; ^c^P < 0:05 for ≥ 12 years vs. < 3 year; ^d^P < 0:05 for 6-11 year vs. 3-5 year; ^e^P < 0:05 for ≥ 12 years vs. 3-5 year; ^f^P < 0:05 for ≥ 12 years vs. 6-11 year.
